# Association between hemoglobin variability and incidence of hypertension over 40 years: a Korean national cohort study

**DOI:** 10.1038/s41598-020-69022-x

**Published:** 2020-07-21

**Authors:** Minkook Son, Junyong Park, Kyungil Park, Sung Yang

**Affiliations:** 1 Department of Biomedical Science and Engineering, Gwangju Institute of Science and Technology, 123 Cheomdan-gwagiro, Buk-gu, Gwangju, 61005 Republic of Korea; 2 School of Mechanical Engineering, Gwangju Institute of Science and Technology, 123 Cheomdan-gwagiro, Buk-gu, Gwangju, 61005 Republic of Korea; 3 Division of Cardiology, Department of Internal Medicine, Dong-A University College of Medicine, Daesingongwon 26, Seo-gu, Busan, 49201 Republic of Korea

**Keywords:** Cardiology, Diseases, Medical research, Risk factors

## Abstract

Hemoglobin level determines blood viscosity and as hemoglobin level rises, blood pressure rises. However, hemoglobin level in individuals is not fixed and change in hemoglobin is affected by various clinical conditions. The purpose of this study is to investigate whether the hemoglobin variability affects the development of hypertension using Korean cohort database. This study was conducted with 94,798 adults (age ≥ 40 years) who visited the health screening in 2006 or 2007 (index year) and had at least 3 health screenings from 2002 to 2007. Hemoglobin variability was assessed by 3 indices of coefficient of variation (CV), standard deviation, and variability independent of the mean. Cox proportional hazard regression analysis was performed for each index of quartile groups (Q1–Q4). A total of 29,145 participants (30.7%) had the incidence of hypertension during a median follow-up of 7.4 ± 2.5 years. In the multivariable adjusted model, the hazard ratio and 95% confidence interval for incidence of hypertension of Q2, Q3, and Q4 compared with Q1 of hemoglobin variability CV were 1.014 [0.981–1.047], 1.064 [1.030–1.099] and 1.094 [1.059–1.131] respectively. The results were consistent in various sensitivity and subgroup analyses. This study showed that hemoglobin variability could be associated with hypertension development.

## Introduction

Hypertension is one of the major risk factors for cardiovascular diseases. It is known that 54% of cerebral infarction and 47% of myocardial infarction are caused by hypertension^1^. Especially, about 60% of cardiovascular diseases in Asia are contributed by hypertension^2,3^. Therefore, lowering blood pressure is considered to reduce the risk of cardiovascular diseases^4^. Hypertension is a multifactorial disease caused by various environmental and genetic factors. Aging, obesity, drinking, and lack of exercise are regarded as risk factors for hypertension^5^. Identifying the potential risk factors and predicting hypertension can help us understand the physiological mechanisms associated with blood pressure, improve the treatment of hypertension, and prevent hypertension.

Hemoglobin is an important factor in determining the viscosity of blood^6^, and several studies reported that as hemoglobin level rises, systolic and diastolic blood pressure rise^7,8^. Hemoglobin level is also associated with arterial stiffness^9^. In animal studies, it is also demonstrated that mean arterial pressure increases with increasing the infusion rate of cross-linked hemoglobin during cardiopulmonary bypass surgery^10^. Besides, it is suggested that the injection of erythropoietin for the treatment of anemia in patients with end-stage renal disease is associated with elevated blood pressure^11^. Although many studies investigated the relationship between blood pressure and hemoglobin levels measured at a one-time point, hemoglobin level in individuals is not fixed and change in hemoglobin is affected by various clinical conditions or diseases^12^. However, no study of the relationship between blood pressure and hemoglobin variability has been reported. The purpose of this study is to investigate whether the hemoglobin variability affects the development of hypertension using large-scale Korean cohort data of the National Health Insurance Service-Health Screening (NHIS-HealS) database.

## Methods

### Data source and study population

This study was approved by the Institutional Review Board of Gwangju Institute of Science and Technology (20190902-EX-02-02) and was performed based on the Declaration of Helsinki. Since the NHIS-Heals data was provided anonymously according to strict confidentiality guidelines, this study was exempted from receiving the subject's informed consent by Institutional Review Board of Gwangju Institute of Science and Technology.

The NHIS database is registered with 98% of Koreans and includes all insurance claims. We analyzed the data using the health screening cohort, which was extracted from a random sampling of approximately 10% of a total 5 million people over 40 years received a health screening in 2002 or 2003. The NHIS provided a mandatory biennial general health screening for people over 40 years. The NHIS-HealS database includes demographic factors (sex, age, and income), hospital utilization (information about the use of medical facilities, admission date, and discharge date), and biennial health screening (height, weight, blood pressure, and blood tests). More information about the database can be found on the relevant website^13^. The NHIS-HealS database has been used in many epidemiological studies and its validity is demonstrated elsewhere^14^.

**Figure 1:**
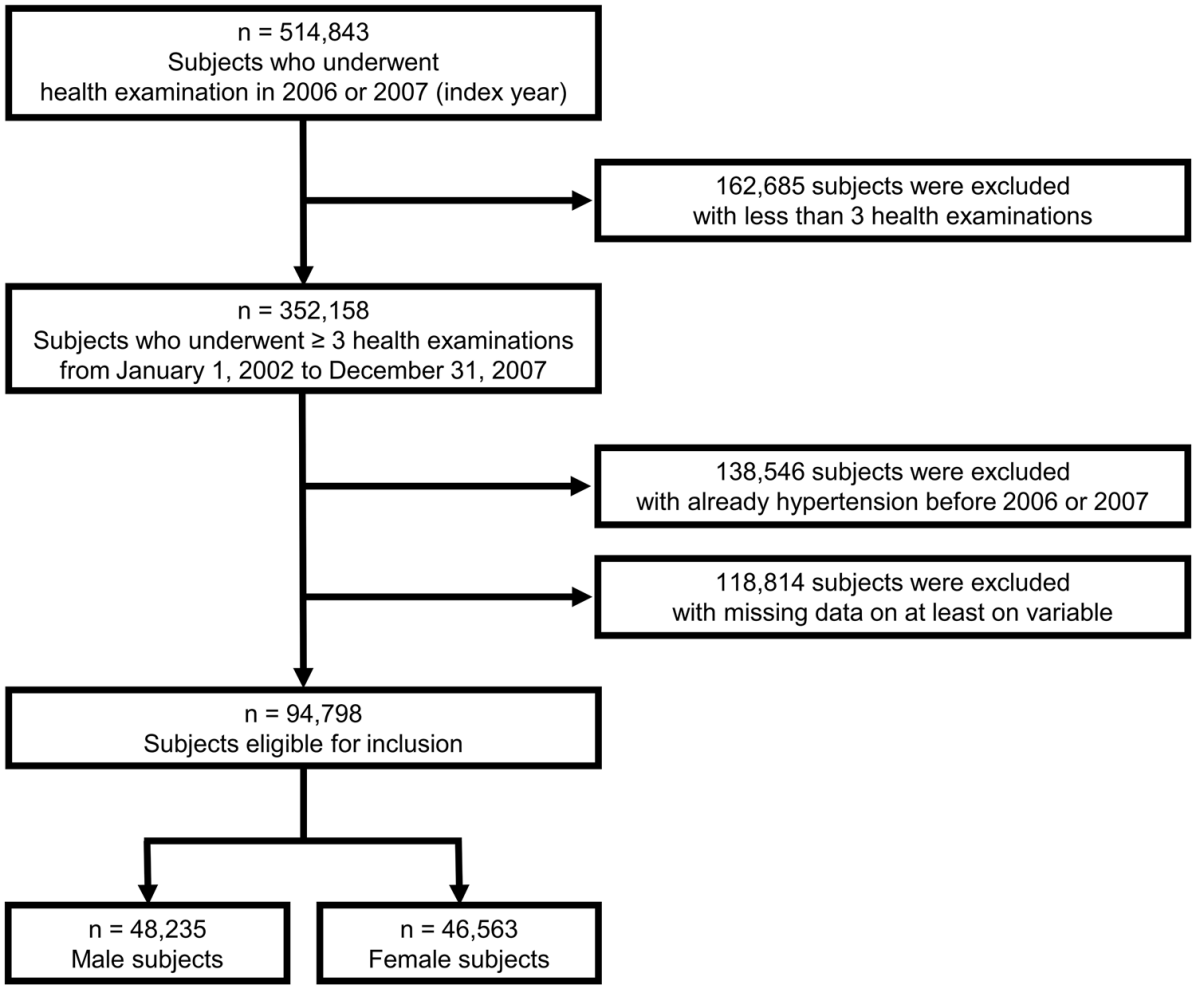
Flow chart of the study population.

This study was conducted with adults (age ≥ 40 years) who visited the health screening in 2006 or 2007 (index year) and had at least 3 health screenings from 2002 to 2007. Subjects meeting the criteria for the diagnosis of hypertension before the index year were excluded. Subjects with one or more missing values were also excluded. Finally, 94,798 subjects (male: 48,235, female: 46,563) were included in this study (Fig. 1).

### Definition of hemoglobin variability

Hemoglobin variability was defined as the variability in measured hemoglobin levels for independent measurements of health screenings. The 3 indicators of hemoglobin variability were used: coefficient of variation (CV), standard deviation (SD), and variability independent of the mean (VIM). CV was calculated as SD × 100/mean and VIM was calculated as SD × 100/mean^beta^. Beta is the regression coefficient based on the natural logarithm of SD over the natural logarithm of the mean^15^. These indicators have already been used in many studies and have been proved to be valid^16–18^. The frequency of hemoglobin measurements ranged from 3 to 6, with 63,099 in 3 measurements, 8,632 in 4 measurements, 9,964 in 5 measurements, and 13,103 in 6 measurements.

### Study outcomes

The endpoint of this study was defined as the incidence of hypertension. Criteria for hypertension were established using the criteria from previous studies^16–18^. The presence of hypertension was defined as the following criteria: (1) the 10th revision of the International Statistical Classification of Diseases and Related Health Problems (ICD-10) codes for hypertension (I10, I11) with at least one claim per year for prescription of the anti-hypertensive drug, or (2) systolic blood pressure (SBP) ≥ 140 mmHg or diastolic blood pressure (DBP) ≥ 90 mmHg. The main analyses were carried out with the above definition, and in addition to the hypertension criteria proposed by the Eighth Joint National Committee (JNC8) guidelines, we performed additional analysis on the hypertension criteria (SBP ≥ 130 mmHg or DBP ≥ 80 mmHg) recently proposed by the American College of Cardiology/American Heart Association (ACC/AHA). Furthermore, for sensitivity analysis, we used another definition of hypertension which includes ICD-10 codes for hypertension with admission ≥ 1 or outpatient department visit ≥ 2, and at least one prescription of anti-hypertensive drug per year^19^. The study population was followed from the day of health screening at index year to the date of incidence of hypertension, the date of death, or December 31, 2015, whichever came first. The follow-up duration was calculated as the difference between the date of outcome and the date of health screening at index year.

### Measurements and definitions

Body mass index (BMI) was calculated by dividing body weight (kg) by the square meter of height (m^2^). Information on smoking status, alcohol consumption, regular exercise, and family history of hypertension was obtained through questionnaires. Smoking status was classified as non-smokers, ex-smokers, and smokers. Drinking status was classified as non-drinking, < 3, and ≥ 3 times per week. Regular exercise was defined as at least five exercises per week. Income level was divided into quintile groups. Samples for the blood test of fasting glucose and total cholesterol were collected after overnight fasting. Hemoglobin variability can be affected by renal function^20^, but the NHIS database doesn’t have creatinine information until 2008. As an alternative, we used urine protein as a confounder. Urine protein was measured with clean, midstream urine by dipstick. It was reported with the following grades: absent, trace, + 1 to + 4. Urine protein was classified as absent, trace, and + 1 or higher in this study^21^. In 2009, creatinine information can be used, and we calculated glomerular filtration rate (GFR) by Modification of Diet in Renal Disease (MDRD) equation^22^. We used this GFR as a confounder in one of the sensitivity analyses. The mean hemoglobin level was calculated by measured hemoglobin levels at health screenings.

The presence of diabetes was defined as the following criteria^16–18^: (1) ICD-10 codes for diabetes (E10–E14) with at least one claim per year for prescription of an anti-diabetic drug, or (2) fasting glucose level ≥ 126 mg/dL. The presence of dyslipidemia was defined as the following criteria^16–18^: (1) ICD-10 codes (E78) for dyslipidemia with at least one claim per year for prescription of lipid-lowering agents or (2) total cholesterol level ≥ 240 mg/dL. We included information about NSAID because Use of NSAID can change hemoglobin and increase blood pressure^23^. The NSAID user was defined with at least one claim per year for prescription of NSAID^24,25^. The Charlson comorbidity index (CCI) was calculated based on the preexisting disease including myocardial infarction, congestive heart failure, peripheral vascular disease, cerebrovascular disease, dementia, chronic pulmonary disease, connective tissue disease, peptic ulcer, mild liver disease, diabetes with and without complications, paraplegia or hemiplegia, renal disease, any or metastatic cancer, moderate or severe liver disease, and acquired immune deficiency syndrome before the start of follow-up period^26,27^.

### Statistical analysis

Baseline characteristics are presented as mean with standard deviation for continuous variables and as number with percentage (%) for categorical variables. The subjects of this study were divided into 4 groups and 10 groups according to hemoglobin variability indices (CV, SD, VIM). Statistical analysis was performed for each indicator in the quartile groups (Q1–Q4) and decile groups (D1–D10). In addition, we performed additional analyses by dividing male and female subjects. The incidence rate of hypertension was calculated by dividing the number of hypertension cases to the total follow-up duration (person-years). The graphs for the incidence rate of hypertension according to the quartiles of hemoglobin variability were calculated using Kaplan–Meier curves and evaluated by the log-rank test. The hazard ratio (HR) and the 95% confidence interval (CI) for the incidence of hypertension were evaluated using the Cox proportional hazards model. The proportional hazard assumption was evaluated by Schoenfeld residuals test with the logarithm of cumulative hazards function based on Kaplan–Meier estimates. There was no interference with the assumption of proportional hazard risk over time. The multivariable-adjusted cox proportional hazard models were adjusted for age, sex, BMI, urine protein, mean hemoglobin level, CCI category, diabetes, dyslipidemia, NSAID use, smoking, exercise, income and family history of hypertension.

Sensitivity analysis was performed as follows: First, we performed the analysis with another definition of hypertension^19^. Second, the additional sensitivity analysis was performed with hypertension criteria recently proposed by the ACC/AHA. Third, we analyzed with participants who received 4 or more health screenings between 2002 and 2009. Fourth, since creatinine information was available in 2009, we conducted the analysis using GFR data with participants in 2009. Fifth, as hemoglobin can be affected in various clinical situations, we conducted the analysis with subjects who didn’t have the hematopoietic disorder, cancer, chronic kidney disease for the study period^28^. Sixth, to investigate the effects of hemoglobin variability in the healthy participants, we performed the additional analysis with CCI = 0 participants. Seventh, we performed the analysis with the absent of urine protein participants to exclude renal effect to hemoglobin variability. Eighth, we also performed the analysis with GFR ≥ 90 participants at the final health screening during the follow-up period. Finally, to exclude the influence of the number of hemoglobin measurements, further analysis was performed with a fixed number of hemoglobin measurements (n = 3).

The stratified analyses were performed to evaluate the potential effect modification by BMI, presence of anemia, family history of hypertension, presence of comorbidities, presence of diabetes, and use of NSAID. In subgroup analysis, we divided 2 subgroups of BMI based on the definition of obesity (BMI ≥ 25 kg/m^2^). The anemia was defined as hemoglobin < 13 g/dL for male and hemoglobin < 12 g/dL for female, as suggested by World Health Organization (WHO) and the presence of comorbidities was defined as the case where CCI is not 0. Furthermore, hemoglobin level can be affected by menstruation, and a stratified analysis was conducted in female subjects dividing by 50 years. Statistical analyses were performed using SAS version 9.4 (SAS Institute Inc., Cary, NC, USA) and using nonlinear regression analysis (PROC NLIN) in SAS, VIM was determined^15^. All figures were produced by R 3.6.0 (https://www.r-project.org/). The *p* value < 0.05 was considered to be statistically significant.

## Results

### Baseline characteristics

The characteristics of the participants divided into 4 groups according to hemoglobin variability CV are shown in Table 1. As the hemoglobin variability increased, the proportion of male gender and current smoker decreased. As the hemoglobin variability increased, the proportion of high CCI category and lower income increased. Mean hemoglobin levels decreased from Q1 to Q4, and the mean CVs in the 4 groups were 1.9%, 3.5%, 5.2%, and 9.7%, respectively. Baseline characteristics of hemoglobin variability SD and VIM are described at Supplementary Table S1. Besides, we divided all subject to male and female and further analyzed. Baseline characteristics in male and female subjects of hemoglobin variability CV are described at Supplementary Table S2.

**Table 1:** Baseline characteristics of all subjects according to the quartiles of hemoglobin variability (CV).

Total (n = 94,798)	Q1 (n = 23,705)	Q2 (n = 23,691)	Q3 (n = 23,702)	Q4 (n = 23,700)
Age (years)	53.4 ± 7.6	53.1 ± 7.4	53.5 ± 7.6	53.7 ± 8.0
Sex (male %)	12,889 (54.3)	13,171 (55.6)	12,299 (51.9)	9,876 (41.7)
BMI (kg/m^2^)	23.4 ± 2.6	23.3 ± 2.6	23.2 ± 2.6	23.1 ± 2.7
Systolic BP (mmHg)	116.5 ± 11.1	116.6 ± 11.1	116.5 ± 11.1	116.3 ± 11.2
Diastolic BP (mmHg)	73.0 ± 7.8	73.1 ± 7.8	73.0 ± 7.8	72.7 ± 7.8
Fasting glucose (mg/dL)	94.5 ± 19.1	94.5 ± 20.4	94.3 ± 20.6	94.4 ± 22.3
Total cholesterol (mg/dL)	197.5 ± 35.1	197.0 ± 35.0	197.3 ± 35.8	195.9 ± 36.3
Urine protein (≥ + 1)	221 (0.9)	241 (1.0)	254 (1.1)	282 (1.2)
Mean hemoglobin (g/dL)	14.0 ± 1.3	14.0 ± 1.3	13.8 ± 1.2	13.3 ± 1.5
**Hemoglobin variability**				
SD (g/dL)	0.3 ± 0.1	0.5 ± 0.1	0.7 ± 0.1	1.3 ± 0.5
CV (%)	1.9 ± 0.7	3.5 ± 0.4	5.2 ± 0.6	9.7 ± 4.3
VIM (%)	4.5 ± 1.8	8.5 ± 1.8	12.2 ± 2.6	20.2 ± 7.2
**CCI category**				
0	15,488 (65.3)	15,452 (65.3)	15,143 (63.9)	14,635 (61.8)
1	5,869 (24.8)	5,907 (24.7)	6,024 (25.3)	6,100 (25.6)
2	1,631 (6.9)	1,624 (7.0)	1,777 (7.5)	2,014 (8.5)
3 ≥	717 (3.0)	708 (3.0)	758 (3.3)	951 (4.1)
Diabetes	1,194 (5.0)	1,152 (4.9)	1,293 (5.5)	1,492 (6.3)
Dyslipidemia	5,642 (23.8)	5,766 (24.3)	5,970 (25.2)	5,893 (24.9)
NSAID user	14,903 (62.9)	14,891 (62.9)	15,256 (64.4)	15,578 (65.7)
Current smoker	4,423 (18.7)	4,647 (19.6)	4,424 (18.7)	3,546 (15.0)
Alcohol consumption (≥ 3 times per week)	1,646 (6.9)	1,716 (7.2)	1,694 (7.1)	1,602 (6.8)
Regular exercise (≥ 5 times per week)	2,264 (9.6)	2,155 (9.1)	2,208 (9.3)	2,146 (9.1)
Income (lower first quintile)	3,463 (14.6)	3,835 (16.2)	4,372 (18.4)	5,053 (21.3)
Family history of hypertension	1,813 (7.7)	1,790 (7.6)	1,694 (7.2)	1,730 (7.3)

Data are expressed as the mean ± SD, or n (%).

*CV* coefficient of variation, *BMI* body mass index, *BP* blood pressure, *SD* standard deviation, *VIM* variability independent of the mean, *CCI* Charlson comorbidity index.

### Association between hemoglobin variability and incidence of hypertension

**Figure 2:**
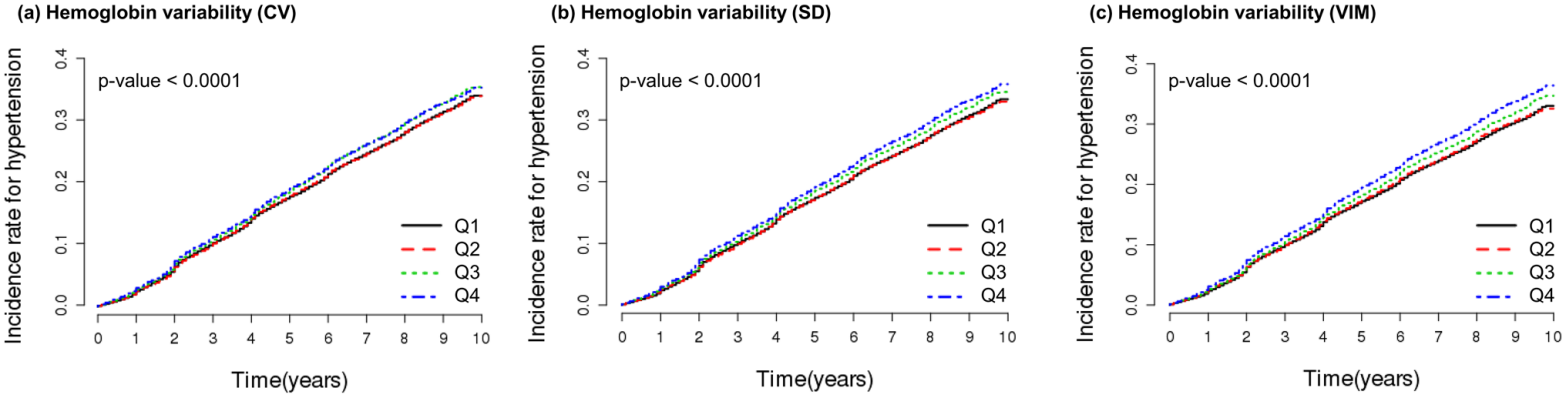
Kaplan–Meier estimates of incidence rate for hypertension by the quartiles of hemoglobin variability. *p* value for Log-rank test. *CV* coefficient of variation, *SD* standard deviation, *VIM* variability independent of the mean, *Q* quartile.

**Figure 3:**
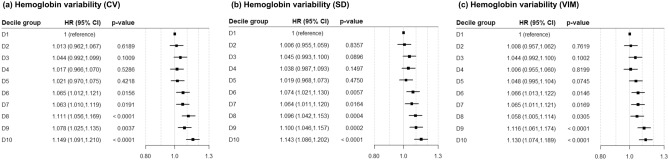
Hazard ratio and 95% confidence interval for incidence of hypertension by the deciles of hemoglobin variability. Adjusted for age, sex, BMI, urine protein, mean hemoglobin level, CCI category, diabetes, dyslipidemia, NSAID use, smoking, exercise, income, and family history of hypertension. *BMI* body mass index, *CCI* Charlson comorbidity index, *CV* coefficient of variation, *SD* standard deviation, *VIM* variability independent of the mean, *D* decile.

A total of 29,145 participants (30.7%) had the incidence of hypertension during a median follow-up of 7.4 ± 2.5 years. Kaplan–Meier curves and log-rank tests showed that the incidence rate of hypertension was higher in the higher quartiles (Fig. 2). The incidence rate of hypertension increased in Q3 and Q4 groups than Q1 and Q2 groups at all of hemoglobin variability indices. Hemoglobin variability and incidence of hypertension were associated in multivariable-adjusted Cox proportional hazard models regardless of gender at all hemoglobin variability indices (Table 2, Supplementary Table S3-S4). In particular, the HR and 95% CIs of Q2, Q3, and Q4 in comparison with Q1 of hemoglobin variability CV in the adjusted model at all subjects were as follows (Q2: 1.014 [0.981–1.047], Q3: 1.064 [1.030–1.099], Q4: 1.094 [1.059–1.131]). This trend was observed in SD and VIM as well. The adjusted HR and 95% CI of female subjects were slightly higher than those of male subjects. We also investigated the HR and 95% CIs of the decile group comparing with D1 as a reference group in the adjusted model to determine the linear trends of the risk. As results, statistical significance was detected in D6 of all hemoglobin variability indices (Fig. 3).

**Table 2:** Hazard ratio and 95% confidence interval for incidence of hypertension according to the quartiles of hemoglobin variability (CV).

Hemoglobin variability (CV)	Events (n)	Follow-up duration (person-years)	Incidence rate (per 1,000 person-years)	HR (95% CI)			
				Unadjusted	*p* value	Adjusted^a^	*p* value
**All subjects (n = 94,798)**							
Q1	7,159	176,418.3	40.58	1		1	
Q2	7,085	175,389.7	40.40	0.996 (0.964, 1.029)	0.8091	1.014 (0.981, 1.047)	0.4189
Q3	7,453	173,491.0	42.96	1.060 (1.026, 1.095)	0.0004	1.064 (1.030, 1.099)	0.0002
Q4	7,448	173,155.5	43.01	1.061 (1.027, 1.096)	0.0003	1.094 (1.059, 1.131)	< 0.0001
*p* for trend				< 0.0001		< 0.0001	
**Male subjects (n = 48,235)**							
Q1	3,793	88,846.6	42.69	1		1	
Q2	3,686	88,490.8	41.65	0.997 (0.934, 1.022)	0.3164	0.993 (0.950, 1.039)	0.7701
Q3	3,893	87,272.4	44.61	1.047 (1.002, 1.095)	0.0424	1.036 (0.991, 1.083)	0.0885
Q4	4,135	85,766.3	48.21	1.133 (1.084, 1.184)	< 0.0001	1.084 (1.037, 1.133)	0.0003
*p* for trend				< 0.0001		< 0.0001	
**Female subjects (n = 46,563)**							
Q1	3,352	87,613.8	38.26	1		1	
Q2	3,391	87,158.3	38.91	1.016 (0.969, 1.066)	0.5050	1.041 (0.992, 1.091)	0.1006
Q3	3,506	86,459.2	40.55	1.060 (1.011, 1.112)	0.0151	1.083 (1.033, 1.136)	0.0009
Q4	3,389	86,847.1	39.02	1.020 (0.972, 1.070)	0.4230	1.118 (1.065, 1.173)	< 0.0001
*p* for trend				0.1863		< 0.0001	

^a^Adjusted for age, sex, BMI, urine protein, mean hemoglobin level, CCI category, diabetes, dyslipidemia, NSAID use, smoking, exercise, income and family history of hypertension.

*CV* coefficient of variation, *BMI* body mass index, *CCI* Charlson comorbidity index, *Q* quartile, *HR* hazard ratio, *CI* confidence interval.

### Sensitivity analysis

The first sensitivity analysis was conducted with another definition of hypertension, and the significant results were observed in all, male and female subject groups (Supplementary Table S5). In the second analysis, we analyzed data with the definition of hypertension (SBP ≥ 130 mmHg or DBP ≥ 80 mmHg) proposed by the ACC/AHA (Supplementary Table S6). The HR and 95% CIs of Q2, Q3, and Q4 in comparison with the Q1 of hemoglobin variability CV in the adjusted model were prominent as follows (Q2: 1.085 [1.035–1.140], Q3: 1.117 [1.064–1.174], Q4: 1.201 [1.142–1.262]). The third sensitivity analysis was performed for subjects who received more than 4 health screenings from 2002 to 2009, and the significant results were also observed in all of hemoglobin variability indices (Supplementary Table S7). The HR and 95% CIs of Q2, Q3, and Q4 in comparison with the Q1 of hemoglobin variability CV in the adjusted model were shown as follows (Q2: 0.980 [0.937–1.025], Q3: 1.051 [1.005–1.099], Q4: 1.117 [1.067–1.168]). The fourth analysis was conducted with GFR information on the subjects who receive the health screening in 2009 (Supplementary Tables S8). The results of hemoglobin variability CV in the adjusted model were more prominent as follows (Q2: 1.013 [0.952–1.077], Q3: 1.102 [1.037–1.172], Q4: 1.176 [1.104–1.253]). The fifth analysis was performed with subjects who didn’t have the hematopoietic disorder, cancer, chronic kidney disease (Supplementary Table S9). The significant results were observed in all of the hemoglobin variability indices. The sixth analysis for participants with CCI = 0 was performed (Supplementary Table S10). The HR and 95% CIs of Q2, Q3, and Q4 in comparison with the Q1 of hemoglobin variability CV in the adjusted model were shown as follows (Q2: 0.990 [0.949–1.032], Q3: 1.060 [1.017–1.105], Q4: 1.084 [1.038–1.131]). The seventh analysis for participants with absent of urine protein was performed (Supplementary Table S11). The HR and 95% CIs of Q2, Q3, and Q4 in comparison with the Q1 of hemoglobin variability CV in the adjusted model were shown as follows (Q2: 1.007 [0.974–1.040], Q3: 1.063 [1.029–1.098], Q4: 1.087 [1.051–1.123]). The eighth analysis for participants with GFR ≥ 90 at the final health screening during the follow-up period was performed (Supplementary Table S12). The significant results were also observed in all of the hemoglobin variability indices. Furthermore, the number of health screenings could act as a confounder, we performed the statistical analysis with the fixed number of hemoglobin measurements (n = 3). The HR and 95% CIs of Q2, Q3, and Q4 in comparison with the Q1 of hemoglobin variability CV in the adjusted model were shown as follows (Q2: 1.007 [0.968–1.047], Q3: 1.061 [1.021–1.103], Q4: 1.083 [1.042–1.124]).

### Subgroup analysis

**Figure 4:**
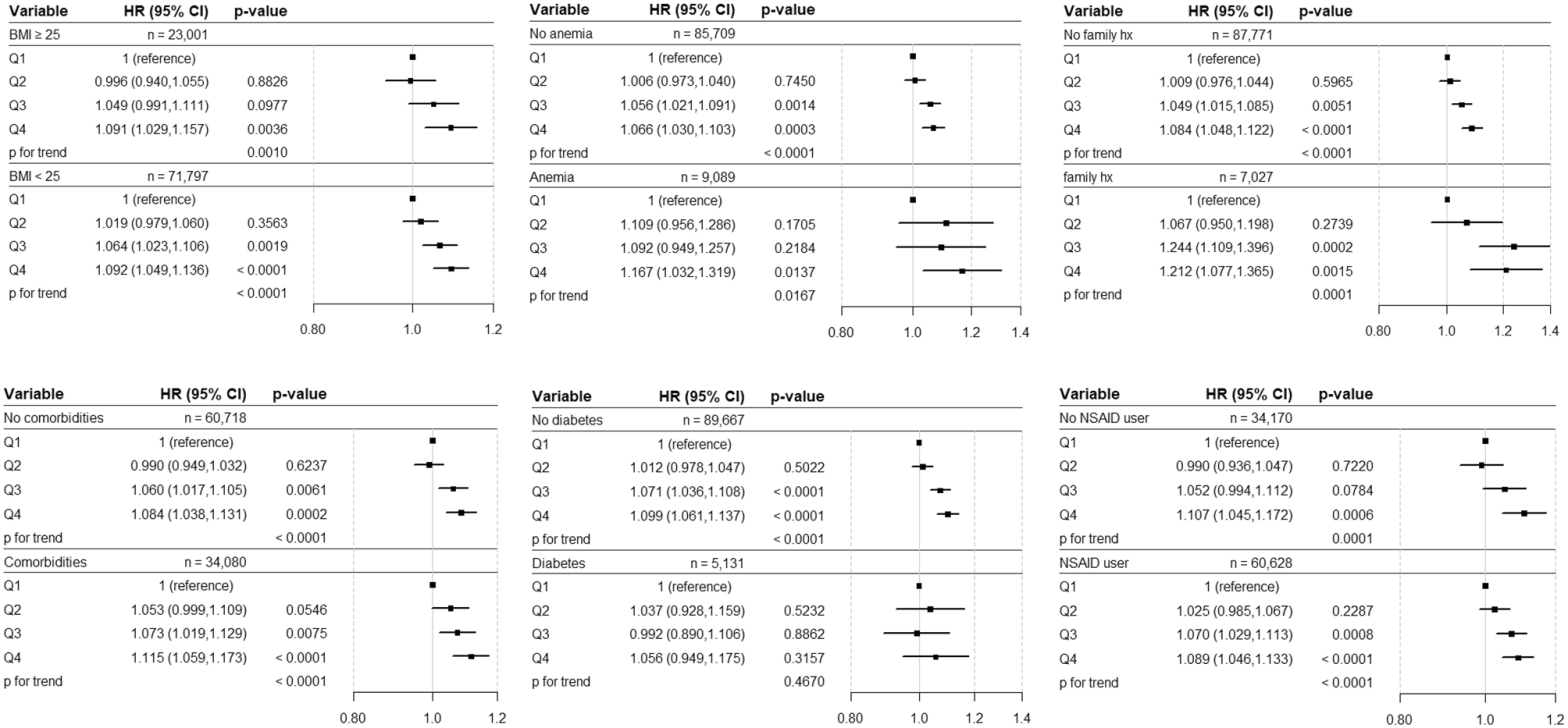
Hazard ratio and 95% confidence interval for incidence of hypertension in the stratified analysis according to the quartiles of hemoglobin variability (CV). Adjusted for age, sex, BMI, urine protein, mean hemoglobin level, CCI category, diabetes, dyslipidemia, NSAID use, smoking, exercise, income, and family history of hypertension. *CV* coefficient of variation, *BMI* body mass index, *CCI* Charlson comorbidity index, *HR* hazard ratio, *CI* confidence interval, *Q* quartile, *Hx* history.

Stratified analyses were performed according to BMI, presence of anemia, family history of hypertension, presence of comorbidities, presence of diabetes, and use of NSAID (Fig. 4). As a result, there were marked increases of HR from Q1 to Q4, below 25 kg/m^2^ in BMI, with anemia, having the family history of hypertension, having comorbidities, and no NSAID user. In stratified analyses according to the presence of diabetes, the HR has a more trend in the subgroup without diabetes. Because of menstruation, the stratified analysis was performed with dividing female subjects by 50 years (Supplementary Figure S1). The HR for the incidence of hypertension of Q4 is higher than that of Q1 at all female subgroups.

## Discussion

This study showed the relationship between hemoglobin variability and the incidence of hypertension through a nationwide population-based cohort study. To the best of our knowledge, there has been no study discussing the association between hemoglobin variability and development of hypertension. Previous studies found the association between hemoglobin level and hypertension, confirming that blood pressure increases as the hemoglobin level rises^7,8^. Kawamoto et al.^29^ reported that hemoglobin level correlates with the pulse wave velocity which is related to the arterial stiffness, leading to elevated blood pressure. Through this study, hemoglobin variability is also associated with the development of hypertension. Our results demonstrated that the incidence of hypertension was higher in the higher hemoglobin variability group than in the lower hemoglobin variability group regardless of hypertension diagnosis criteria of JNC and ACC/AHA guideline (Table 2, Supplementary Table S6). Furthermore, the results were valid when the number and period for measurements of hemoglobin levels (6 years for the main study) were extended (Supplementary Table S7). Hemoglobin variability is affected by renal function, we analyzed the subjects with GFR information (Supplementary Table S8), the subject with absent of urine protein (Supplementary Table S11), and the subject with GFR ≥ 90 at the final health screening during the follow-up period (Supplementary Table S12). When analyzed for participants with CCI = 0, the results were consistent (Supplementary Table S10). In the stratified analysis, most of the results were consistent with our main findings (Fig. 4).

The concept of hemoglobin variability comes from patients of end-stage renal disease, who are susceptible to anemia^20^. There are many methods to define hemoglobin variability. However, with no gold standard to measure variability, we used 3 representative indices of variability based on various studies that refer to variability^15–18^. SD is the most widely used indicator and used in this study. As SD can be affected by the mean^30^, CV and VIM, where SD is adjusted to mean, were also used. We evaluated the effect of hemoglobin variability on hypertension development with the mentioned 3 indices (CV, SD, and VIM) and verified that the results were all valid.

The exact mechanism that explains this result is not yet clearly identified, however, some hypotheses may be suggested. First of all, the hemoglobin level can be considered as a biomarker that reflects the inflammatory state of the body. Red blood cell distribution width (RDW) is suggested as an inflammatory marker that can worsen the arteriosclerosis and RDW is related to cardiovascular disease^31,32^. Furthermore, RDW is higher in patients with hypertension than in healthy persons^33^. The systemic chronic inflammation hinders the activity of bone marrow, reducing the production of red blood cell^12^. Thus, the inflammatory condition not only increases RDW but also can cause changes in hemoglobin level, and inflammation can lead to atherosclerosis as a principal pathophysiological inducer^34^. Similarly, high hemoglobin variability is thought to be a sign of inflammation and oxidative stress, which are important factors underlying hypertension^35,36^.

In addition, hemoglobin itself is a factor that can affect blood pressure. Firstly, Vázquez et al.^37^ reported that a rapid decrease in 10% of hematocrit can raise the blood pressure of 8.6 mmHg and long-term hematocrit change can have the same tendency as acute change has. Hematocrit can affect blood pressure as it determines blood viscosity and regulates peripheral vascular resistance^38^. Christine et al.^39^ suggested that red blood cell affects the function of endothelial nitric oxide synthase (eNOS) and smooth muscle contractility through regulation of oxygen transport. Ganidagli et al.^40^ presented that the carotid intima-media thickness, which is the result of atherosclerosis, was significantly higher in higher hemoglobin variability group among hemodialysis patients and Orcun et al. described that higher hemoglobin variability group showed a significant increase on left ventricular mass index, which is associated with blood pressure^41^. Secondly, hemoglobin has a high affinity with nitric oxide (NO)^42^. Especially, scavenging of NO caused by hemoglobin outside the erythrocytes can cause vasoconstriction^39^. Furthermore, cell-free hemoglobin has pro-oxidant and pro-inflammatory activities^43^. Although the hemoglobin level is affected by various situations, cell-free hemoglobin generated by hemolysis can cause vasoconstriction and hypertension. Therefore, considering the above mechanisms, hemoglobin variability can be associated with the development of hypertension.

There are some limitations of this study. Firstly, inconsistencies between actual medical practices performed on individuals and recorded claims data may lead to inaccurate analysis. To reduce the risk of inaccuracy, we defined hypertension using the definition used in previous studies^16–18^. But, there was the possibility of misclassification bias for hypertension. Secondly, Hemoglobin variability may vary with an individual’s status, and all of these conditions can be potential covariates. However, as we cannot evaluate all kinds of comorbidities, we used CCI, the most widely used comorbidity assessment tool^26,27^. Thirdly, there is the possibility of unadjusted confounders, such as inflammatory markers. Besides, we used the use of NSAID as a covariate, but due to the variety of ingredients and formulations of the drug, we could not consider the dose. Considering these points, further studies are needed. Finally, since this study is conducted only for Koreans, it is needed to perform the additional research to find out whether our results could be applied to other countries.

Despite these limitations, this study has several strengths. This study was a large-scale, long-term follow-up study representing the middle-aged Korean population. The incidence rate of hypertension per 1,000 person-years is about 37 to 44 in the Atherosclerosis Risk In Communities (ARIC) study^44^, which is similar to our results, supporting that our study population was well-established. Through various sensitivity analyses and subgroup analyses, we showed that hemoglobin variability could be related to hypertension development. Hemoglobin test is a routine blood test in health screening and easily identified. Predicting for hypertension with simple periodic health screening results would be a meaningful approach for individuals.

This retrospective cohort study showed the association between hemoglobin variability and incidence of hypertension over 40 years and suggested that the higher hemoglobin variability is correlated with the higher incidence of hypertension. Hemoglobin variability may be a predictor and risk factor for the development of hypertension, but further studies are needed to confirm the exact pathophysiological mechanism for hemoglobin variability and hypertension.

## Supplementary information

Supplementary Information.
