# Supplementary material for: Association between hemoglobin variability and incidence of hypertension over 40 years: a Korean national cohort study
Source: Sci Rep. 2020 Jul 21;10:12061. doi: 10.1038/s41598-020-69022-x (PMC7374722; doi:10.1038/s41598-020-69022-x)

**Association between hemoglobin variability and incidence of hypertension over 40 years: A Korean national cohort study**

**Minkook Son^1^, Junyong Park^1^, Kyungil Park^3*^, and Sung Yang^1,2*^**

**Author Affiliations**

^1^Department of Biomedical Science and Engineering, Gwangju Institute of Science and Technology

^2^School of Mechanical Engineering, Gwangju Institute of Science and Technology

^3^Division of Cardiology, Department of Internal Medicine, Dong-A University College of Medicine

**Corresponding authors**

Kyungil Park, MD, Ph D

E-mail: cardiopark@gmail.com

Sung Yang, Ph.D,

E-mail: syang@gist.ac.kr

**Supplementary materials**

Supplementary Table S1. Baseline characteristics of all subjects according to the quartiles of the hemoglobin variability (SD, VIM).

Supplementary Table S2. Baseline characteristics of male and female subjects according to the quartiles of the hemoglobin variability (CV).

Supplementary Table S3. Hazard ratio and 95% confidence interval for incidence of hypertension in male and female subjects according to the quartiles of hemoglobin variability (SD).

Supplementary Table S4. Hazard ratio and 95% confidence interval in male and female subjects for incidence of hypertension according to the quartiles of hemoglobin variability (VIM).

Supplementary Table S5. Hazard ratio and 95% confidence interval for incidence of hypertension according to the quartiles of hemoglobin variability (CV) by another definition of hypertension.

Supplementary Table S6. Hazard ratio and 95% confidence interval for incidence of hypertension according to the quartiles of hemoglobin variability (Study outcomes with SBP ≥ 130 mmHg or DBP ≥ 80 mmHg, ACC/AHA criteria for hypertension, n = 19,871).

Supplementary Table S7. Hazard ratio and 95% confidence interval for incidence of hypertension according to the quartiles of hemoglobin variability (Subjects with 4 ≥ health screenings in 2008 & 2009 as index year, n = 70,389).

Supplementary Table S8. Hazard ratio and 95% confidence interval for incidence of hypertension according to the quartiles of hemoglobin variability (Subjects with 4 ≥ health screenings in 2009 as index year, n = 39,591)

Supplementary Table S9. Hazard ratio and 95% confidence interval for incidence of hypertension according to the quartiles of hemoglobin variability (Subjects without hematopoietic disorder, cancer, chronic kidney disease, n = 51,490).

Supplementary Table S10. Hazard ratio and 95% confidence interval for incidence of hypertension according to the quartiles of hemoglobin variability (Subjects with CCI = 0, n = 60,718).

Supplementary Table S11. Hazard ratio and 95% confidence interval for incidence of hypertension according to the quartiles of hemoglobin variability (Subjects with absent of urine protein, n = 93,579).

Supplementary Table S12. Hazard ratio and 95% confidence interval for incidence of hypertension according to the quartiles of hemoglobin variability (Subjects with GFR ≥ 90 at the final health screening, n = 43,942).

Supplementary Figure S1. Hazard ratio and 95% confidence interval for incidence of hypertension of hemoglobin variability (CV) in female subjects dividing by 50 years.

**Supplementary Table S1. Baseline characteristics of all subjects according to the quartiles of the hemoglobin variability (SD, VIM)**

**(a) Hemoglobin variability (SD)**

| **Total**  **(n = 94,798)** | **Q1**  **(n = 23,700)** | **Q2**  **(n = 23,698)** | **Q3**  **(n = 23,756)** | **Q4**  **(n = 23,644)** |
| --- | --- | --- | --- | --- |
| Age (years) | 53.5 ± 7.7 | 53.1 ± 7.4 | 53.4 ± 7.6 | 53.6 ± 7.9 |
| Sex (male %) | 11,316 (47.8) | 12,497 (52.7) | 12,647 (53.2) | 11,775 (49.8) |
| BMI (kg/m^2^) | 23.3 ± 2.6 | 23.3 ± 2.6 | 23.2 ± 2.6 | 23.2 ± 2.7 |
| Systolic BP (mmHg) | 116.3 ± 11.2 | 116.5 ± 11.1 | 116.5 ±11.1 | 116.6 ± 11.1 |
| Diastolic BP (mmHg) | 72.7 ± 7.9 | 72.9 ± 7.8 | 73.0 ± 7.8 | 73.0 ± 7.8 |
| Fasting glucose (mg/dL) | 94.0 ± 18.5 | 94.4 ± 20.4 | 94.4 ± 20.3 | 95.0 ± 23.1 |
| Total cholesterol (mg/dL) | 197.4 ± 35.1 | 196.9 ± 35.5 | 197.3 ± 35.4 | 196.0 ± 36.2 |
| Urine protein (≥+1) | 220 (0.9) | 245 (1.0) | 253 (1.1) | 280 (1.2) |
| Mean hemoglobin (g/dL) | 13.8 ± 1.3 | 13.9 ± 1.3 | 13.8 ± 1.3 | 13.6 ± 1.5 |
| **Hemoglobin variability** |  |  |  |  |
| SD (g/dL) | 0.3 ± 0.1 | 0.5 ± 0.1 | 0.7 ± 0.1 | 1.3 ± 0.5 |
| CV (%) | 1.9 ± 0.7 | 3.6 ± 0.5 | 5.2 ± 0.8 | 9.6 ± 4.3 |
| VIM (%) | 4.3 ± 1.6 | 8.3 ± 1.3 | 12.1 ± 1.8 | 20.7 ± 6.8 |
| **CCI category** |  |  |  |  |
| 0 | 15,383 (64.9) | 15,501 (65.4) | 15,194 (64.0) | 14,640 (61.9) |
| 1 | 5,961 (25.2) | 5,833 (24.6) | 6,033 (25.4) | 6,073 (25.7) |
| 2 | 1,642 (6.9) | 1,659 (7.0) | 1,751 (7.4) | 1,994 (8.4) |
| 3 ≥ | 714 (3.0) | 705 (3.0) | 778 (3.2) | 937 (4.0) |
| Diabetes | 1,128 (4.8) | 1,132 (4.8) | 1,301 (5.5) | 1,570 (6.6) |
| Dyslipidemia | 5,600 (23.6) | 5,736 (24.2) | 5,988 (25.2) | 5,947 (25.2) |
| NSAID user | 15,130 (63.8) | 14,974 (63.2) | 15,167 (63.8) | 15,357 (65.0) |
| Current smoker | 3,888 (16.4) | 4,350 (18.4) | 4,589 (19.3) | 4,213 (17.8) |
| Alcohol consumption  (≥3 times per week) | 1,474 (6.2) | 1,635 (6.9) | 1,705 (7.2) | 1,844 (7.8) |
| Regular exercise  (≥5 times per week) | 2,268 (9.6) | 2,180 (9.2) | 2,194 (9.2) | 2,131 (9.0) |
| Income  (lower first quintile) | 3,728 (15.7) | 3,902 (16.5) | 4,324 (18.2) | 4,769 (20.2) |
| family history of hypertension | 1,862 (7.9) | 1,765 (7.5) | 1,705 (7.2) | 1,695 (7.2) |

**(b) Hemoglobin variability (VIM)**

| **Total**  **(n = 94,798)** | **Q1**  **(n = 23,704)** | **Q2**  **(n = 23,695)** | **Q3**  **(n = 23,700)** | **Q4**  **(n = 23,699)** |
| --- | --- | --- | --- | --- |
| Age (years) | 53.7 ± 7.8 | 53.2 ± 7.5 | 53.2 ± 7.5 | 53.5 ± 7.9 |
| Sex (male %) | 9,587 (40.4) | 11,645 (49.2) | 13,099 (55.3) | 13,904 (58.7) |
| BMI (kg/m^2^) | 23.2 ± 2.6 | 23.2 ± 2.6 | 23.3 ± 2.6 | 23.3 ± 2.7 |
| Systolic BP (mmHg) | 115.9 ± 11.3 | 116.3 ± 11.2 | 116.6 ±11.0 | 117.0 ± 11.0 |
| Diastolic BP (mmHg) | 72.4 ± 7.9 | 72.8 ± 7.8 | 73.1 ± 7.8 | 73.3 ± 7.7 |
| Fasting glucose (mg/dL) | 93.7 ± 18.3 | 94.1 ± 19.6 | 94.5 ± 20.3 | 95.5 ± 23.8 |
| Total cholesterol (mg/dL) | 197.3 ± 35.3 | 196.9 ± 35.5 | 197.3 ± 35.6 | 196.1 ± 35.9 |
| Urine protein (≥+1) | 225 (1.0) | 241 (1.0) | 236 (1.0) | 296 (1.3) |
| Mean hemoglobin (g/dL) | 13.5 ± 1.3 | 13.8 ± 1.3 | 13.9 ± 1.3 | 13.9 ± 1.5 |
| **Hemoglobin variability** |  |  |  |  |
| SD (g/dL) | 0.3 ± 0.1 | 0.5 ± 0.1 | 0.7 ± 0.1 | 1.3 ± 0.5 |
| CV (%) | 2.0 ± 0.8 | 3.7 ± 0.9 | 5.3 ± 1.3 | 9.3 ± 4.5 |
| VIM (%) | 4.3 ± 1.5 | 8.2 ± 1.0 | 12.1 ± 1.3 | 20.8 ± 6.7 |
| **CCI category** |  |  |  |  |
| 0 | 15,282 (64.5) | 15,472 (65.3) | 15,195 (64.1) | 14,769 (62.3) |
| 1 | 6,021 (25.4) | 5,896 (24.7) | 5,968 (25.2) | 6,015 (25.4) |
| 2 | 1,675 (7.1) | 1,631 (7.1) | 1,766 (7.5) | 1,974 (8.3) |
| 3 ≥ | 726 (3.0) | 696 (2.9) | 771 (3.2) | 941 (4.0) |
| Diabetes | 1,110 (4.7) | 1,067 (4.5) | 1,299 (5.5) | 1,655 (7.0) |
| Dyslipidemia | 5,575 (23.5) | 5,731 (24.2) | 5,970 (25.2) | 5,995 (25.3) |
| NSAID user | 15,407 (65.0) | 15,042 (63.5) | 15,004 (63.3) | 15,175 (64.0) |
| Current smoker | 3,246 (13.7) | 4,031 (17.0) | 4,751 (20.1) | 5,012 (21.2) |
| Alcohol consumption  (≥3 times per week) | 1,646 (6.9) | 1,716 (7.2) | 1,694 (7.1) | 1,602 (6.8) |
| Regular exercise  (≥5 times per week) | 2,321 (9.8) | 2,145 (9.1) | 2,150 (9.1) | 2,157 (9.1) |
| Income  (lower first quintile) | 3,463 (14.6) | 3,835 (16.2) | 4,372 (18.4) | 5,053 (21.3) |
| family history of hypertension | 1,905 (8.0) | 1,760 (7.4) | 1,678 (7.1) | 1,684 (7.1) |

Data are expressed as the mean ± SD, or n (%).

SD, standard deviation; VIM, variability independent of the mean; BMI, body mass index; BP, blood pressure; CV, coefficient of variation; CCI, Charlson comorbidity index.

**Supplementary Table S2. Baseline characteristics of male and female subjects according to the quartiles of the hemoglobin variability (CV)**

**(a) Male subjects**

| **Total**  **(n = 48,235)** | **Q1**  **(n = 12,058)** | **Q2**  **(n = 12,059)** | **Q3**  **(n = 12,059)** | **Q4**  **(n = 12,059)** |
| --- | --- | --- | --- | --- |
| Age (years) | 52.8 ± 7.5 | 52.5 ± 7.2 | 53.1 ± 7.6 | 54.5 ± 8.4 |
| BMI (kg/m^2^) | 23.5 ± 2.6 | 23.4 ± 2.6 | 23.4 ± 2.6 | 23.2 ± 2.7 |
| Systolic BP (mmHg) | 117.8 ± 10.6 | 117.8 ± 10.5 | 117.8 ±10.6 | 117.8 ± 10.6 |
| Diastolic BP (mmHg) | 74.3 ± 7.4 | 74.2 ± 7.5 | 74.1 ± 7.5 | 74.0 ± 7.5 |
| Fasting glucose (mg/dL) | 96.2 ± 20.0 | 96.7 ± 22.8 | 96.5 ± 22.5 | 97.3 ± 25.2 |
| Total cholesterol (mg/dL) | 195.2 ± 34.1 | 194.7 ± 34.3 | 194.7 ± 34.5 | 193.1 ± 35.5 |
| Urine protein (≥+1) | 114 (1.0) | 114 (1.0) | 137 (1.1) | 144 (1.2) |
| Mean hemoglobin (g/dL) | 14.9 ± 0.8 | 14.8 ± 0.8 | 14.8 ± 0.8 | 14.5 ± 0.9 |
| **Hemoglobin variability** |  |  |  |  |
| SD (g/dL) | 0.3 ± 0.1 | 0.5 ± 0.1 | 0.7 ± 0.1 | 1.2 ± 0.4 |
| CV (%) | 1.8 ± 0.6 | 3.4 ± 0.4 | 4.9 ± 0.5 | 8.3 ± 3.2 |
| VIM (%) | 5.0 ± 1.8 | 9.1 ± 1.6 | 13.0 ± 2.0 | 21.2 ± 6.6 |
| **CCI category** |  |  |  |  |
| 0 | 8,236 (68.3) | 8,200 (68.0) | 7,940 (65.8) | 7,482 (62.0) |
| 1 | 2,753 (22.8) | 2,750 (22.8) | 2,878 (23.9) | 2,966 (24.6) |
| 2 | 724 (6.0) | 779 (6.5) | 845 (7.0) | 1,055 (8.8) |
| 3 ≥ | 345 (2.9) | 330 (2.7) | 396 (3.3) | 556 (4.6) |
| Diabetes | 717 (6.0) | 707 (5.9) | 796 (6.6) | 997 (8.3) |
| Dyslipidemia | 2,657 (22.0) | 2,726 (22.6) | 2,842 (23.6) | 2,878 (23.9) |
| NSAID user | 6,835 (56.7) | 6,825 (56.6) | 6,961 (57.7) | 7,237 (60.0) |
| Current smoker | 3,991 (33.1) | 4,076 (33.8) | 4,262 (35.3) | 4,105 (34.0) |
| Alcohol consumption  (≥3 times per week) | 1,415 (11.7) | 1,452 (12.0) | 1,518 (12.6) | 1,719 (14.3) |
| Regular exercise  (≥5 times per week) | 1,034 (8.6) | 1,084 (9.0) | 1,115 (9.3) | 1,128 (9.4) |
| Income  (lower first quintile) | 1,114 (9.2) | 1,283 (10.6) | 1,540 (12.8) | 1,823 (15.1) |
| family history of hypertension | 777 (6.4) | 774 (6.4) | 743 (6.2) | 666 (5.5) |

**(b) Female subjects**

| **Total**  **(n = 46,563)** | **Q1**  **(n = 11,641)** | **Q2**  **(n = 11,641)** | **Q3**  **(n = 11,641)** | **Q4**  **(n = 11,640)** |
| --- | --- | --- | --- | --- |
| Age (years) | 54.1 ± 7.7 | 53.7 ± 7.6 | 53.8 ± 7.6 | 52.8 ± 7.5 |
| BMI (kg/m^2^) | 23.2 ± 2.7 | 23.2 ± 2.7 | 23.1 ± 2.7 | 23.0 ± 2.7 |
| Systolic BP (mmHg) | 115.0 ± 11.6 | 115.0 ± 11.6 | 115.2 ±11.5 | 115.1 ± 11.5 |
| Diastolic BP (mmHg) | 71.5 ± 8.0 | 71.5 ± 8.0 | 71.8 ± 8.0 | 71.7 ± 7.9 |
| Fasting glucose (mg/dL) | 92.4 ± 17.5 | 91.9 ± 17.5 | 92.2 ± 17.9 | 92.1 ± 18.9 |
| Total cholesterol (mg/dL) | 200.2 ± 35.9 | 199.7 ± 35.9 | 200.4 ± 36.8 | 197.6 ± 36.7 |
| Urine protein (≥+1) | 108 (0.9) | 128 (1.1) | 129 (1.1) | 130 (1.1) |
| Mean hemoglobin (g/dL) | 12.9 ± 0.7 | 12.9 ± 0.8 | 12.8 ± 0.8 | 12.4 ± 1.1 |
| **Hemoglobin variability** |  |  |  |  |
| SD (g/dL) | 0.2 ± 0.1 | 0.5 ± 0.1 | 0.7 ± 0.1 | 1.3 ± 0.5 |
| CV (%) | 1.9 ± 0.7 | 3.7 ± 0.5 | 5.6 ± 0.7 | 10.9 ± 4.9 |
| VIM (%) | 3.9 ± 1.5 | 7.5 ± 1.3 | 11.2 ± 1.9 | 19.7 ± 7.3 |
| **CCI category** |  |  |  |  |
| 0 | 7,161 (61.5) | 7,304 (62.7) | 7,171 (61.6) | 7,224 (62.1) |
| 1 | 3,197 (27.5) | 3,117 (26.8) | 3,156 (27.1) | 3,083 (26.5) |
| 2 | 912 (7.8) | 862 (7.4) | 925 (8.0) | 944 (8.1) |
| 3 ≥ | 371 (3.2) | 358 (3.1) | 389 (3.3) | 389 (3.3) |
| Diabetes | 441 (3.8) | 428 (3.7) | 505 (4.3) | 540 (4.6) |
| Dyslipidemia | 3,007 (25.8) | 3,069 (26.4) | 3,160 (27.2) | 2,932 (25.2) |
| NSAID user | 8,183 (70.3) | 8,224 (70.7) | 8,241 (70.8) | 8,122 (69.8) |
| Current smoker | 162 (1.4) | 135 (1.2) | 161 (1.4) | 148 (1.3) |
| Alcohol consumption  (≥3 times per week) | 135 (1.2) | 130 (1.1) | 136 (1.2) | 153 (1.3) |
| Regular exercise  (≥5 times per week) | 1,211 (10.4) | 1,120 (9.6) | 1,089 (9.4) | 992 (8.5) |
| Income  (lower first quintile) | 1,214 (10.4) | 1,124 (9.7) | 1,096 (9.4) | 996 (8.6) |
| family history of hypertension | 1,072 (9.2) | 1,017 (8.7) | 951 (8.2) | 1,027 (8.8) |

Data are expressed as the mean ± SD, or n (%).

CV, coefficient of variation; BMI, body mass index; BP, blood pressure; SD, standard deviation; VIM, variability independent of the mean; CCI, Charlson comorbidity index.

**Supplementary Table S3. Hazard ratio and 95% confidence interval for incidence of hypertension in male and female subjects according to the quartiles of hemoglobin variability (SD)**

| **Hemoglobin**  **variability**  **(SD)** | Events (n) | Follow-up duration (person-years) | Incidence rate  (per 1,000  person-years) | **HR (95% CI)** | | | |
| --- | --- | --- | --- | --- | --- | --- | --- |
|  |  |  |  | **Unadjusted** | **p-value** | **Adjusted^*^** | **p-value** |
| **All subjects**  **(n = 94,798)** |  |  |  |  |  |  |  |
| Q1 | 7,103 | 176,684.7 | 40.20 | 1 |  | 1 |  |
| Q2 | 7,028 | 175,753.7 | 39.99 | 0.995 (0.963, 1.028) | 0.7680 | 1.008 (0.976, 1.042) | 0.6323 |
| Q3 | 7,407 | 174,178.6 | 42.53 | 1.059 (1.025, 1.094) | 0.0006 | 1.054 (1.020, 1.089) | 0.0015 |
| Q4 | 7,607 | 171,837.5 | 44.27 | 1.103 (1.068, 1.139) | < 0.0001 | 1.099 (1.064, 1.135) | < 0.0001 |
| p for trend |  |  |  | < 0.0001 |  | < 0.0001 |  |
| **Male subjects**  **(n = 48,235)** |  |  |  |  |  |  |  |
| Q1 | 3,791 | 89,312.2 | 42.45 | 1 |  | 1 |  |
| Q2 | 3,652 | 87,763.5 | 41.61 | 0.982 (0.938, 1.028) | 0.4327 | 0.995 (0.951, 1.041) | 0.8431 |
| Q3 | 3,889 | 87,604.9 | 44.39 | 1.048 (1.002, 1.096) | 0.0387 | 1.034 (0.989, 1.081) | 0.1430 |
| Q4 | 4,175 | 85,695.5 | 48.72 | 1.152 (1.102, 1.204) | < 0.0001 | 1.090 (1.044, 1.139) | < 0.0001 |
| p for trend |  |  |  | < 0.0001 |  | < 0.0001 |  |
| **Female subjects**  **(n = 46,563)** |  |  |  |  |  |  |  |
| Q1 | 3,304 | 86,993.0 | 37.98 | 1 |  | 1 |  |
| Q2 | 3,420 | 88,793.2 | 38.52 | 1.014 (0.966, 1.063) | 0.5802 | 1.033 (0.985, 1.084) | 0.1760 |
| Q3 | 3,457 | 86,055.6 | 40.17 | 1.058 (1.009, 1.109) | 0.0209 | 1.078 (1.028, 1.131) | 0.0019 |
| Q4 | 3,457 | 86,236.6 | 40.09 | 1.056 (1.006, 1.107) | 0.0261 | 1.130 (1.077, 1.185) | < 0.0001 |
| p for trend |  |  |  | 0.0075 |  | < 0.0001 |  |

^*^adjusted for age, sex, BMI, urine protein, mean hemoglobin level, CCI category, diabetes, dyslipidemia, NSAID use, smoking, exercise, income and family history of hypertension.

SD, standard deviation; BMI, body mass index; CCI, Charlson comorbidity index; Q, quartile; HR, hazard ratio; CI, confidence interval.

**Supplementary Table S4. Hazard ratio and 95% confidence interval in male and female subjects for incidence of hypertension according to the quartiles of hemoglobin variability (VIM)**

| **Hemoglobin**  **variability**  **(VIM)** | Events (n) | Follow-up duration (person-years) | Incidence rate  (per 1,000  person-years) | **HR (95% CI)** | | | |
| --- | --- | --- | --- | --- | --- | --- | --- |
|  |  |  |  | **Unadjusted** | **p-value** | **Adjusted^*^** | **p-value** |
| **All subjects**  **(n = 94,798)** |  |  |  |  |  |  |  |
| Q1 | 7,023 | 177,191.7 | 39.64 | 1 |  | 1 |  |
| Q2 | 7,024 | 175,688.9 | 39.98 | 1.009 (0.976, 1.043) | 0.5835 | 1.025 (0.992, 1.059) | 0.1425 |
| Q3 | 7,355 | 174,034.6 | 42.26 | 1.068 (1.033, 1.103) | < 0.0001 | 1.055 (1.021, 1.090) | 0.0014 |
| Q4 | 7,743 | 171,539.3 | 45.14 | 1.141 (1.105, 1.179) | < 0.0001 | 1.101 (1.065, 1.137) | < 0.0001 |
| p for trend |  |  |  | < 0.0001 |  | < 0.0001 |  |
| **Male subjects**  **(n = 48,235)** |  |  |  |  |  |  |  |
| Q1 | 3,787 | 88,812.3 | 42.64 | 1 |  | 1 |  |
| Q2 | 3,684 | 88,325.2 | 41.71 | 0.979 (0.936, 1.025) | 0.9790 | 0.991 (0.948, 1.037) | 0.7004 |
| Q3 | 3,831 | 87,525.2 | 43.77 | 1.029 (0.983, 1.076) | 0.2176 | 1.011 (0.967, 1.057) | 0.6327 |
| Q4 | 4,205 | 85,713.4 | 49.06 | 1.154 (1.105, 1.206) | < 0.0001 | 1.089 (1.042, 1.137) | < 0.0001 |
| p for trend |  |  |  | < 0.0001 |  | < 0.0001 |  |
| **Female subjects**  **(n = 46,563)** |  |  |  |  |  |  |  |
| Q1 | 3,320 | 87,784.0 | 37.82 | 1 |  | 1 |  |
| Q2 | 3,324 | 87,563.4 | 37.96 | 1.003 (0.956, 1.053) | 0.8920 | 1.022 (0.974, 1.072) | 0.3832 |
| Q3 | 3,485 | 86,568.3 | 40.26 | 1.065 (1.015, 1.116) | 0.0099 | 1.081 (1.031, 1.134) | 0.0012 |
| Q4 | 3,509 | 86,162.7 | 40.73 | 1.078 (1.028, 1.130) | 0.0002 | 1.120 (1.068, 1.175) | < 0.0001 |
| p for trend |  |  |  | 0.0002 |  | < 0.0001 |  |

^*^adjusted for age, sex, BMI, urine protein, mean hemoglobin level, CCI category, diabetes, dyslipidemia, NSAID use, smoking, exercise, income and family history of hypertension.

VIM, variability independent of the mean; BMI, body mass index; CCI, Charlson comorbidity index; Q, quartile; HR, hazard ratio; CI, confidence interval.

**Supplementary Table S5. Hazard ratio and 95% confidence interval for incidence of hypertension according to the quartiles of hemoglobin variability (CV) by another definition^‡^ of hypertension**

| **Hemoglobin**  **variability**  **(CV)** | Events (n) | Follow-up duration (person-years) | Incidence rate  (per 1,000  person-years) | **HR (95% CI)** | | | |
| --- | --- | --- | --- | --- | --- | --- | --- |
|  |  |  |  | **Unadjusted** | **p-value** | **Adjusted^*^** | **p-value** |
| **All subjects**  **(n = 162,233)** |  |  |  |  |  |  |  |
| Q1 | 12,438 | 291,679.4 | 42.64 | 1 |  | 1 |  |
| Q2 | 12,316 | 290,105.6 | 42.45 | 0.994 (0.970, 1.019) | 0.6487 | 1.011 (0.986, 1.036) | 0.4048 |
| Q3 | 12,738 | 287,301.8 | 44.34 | 1.037 (1.012, 1.063) | 0.0035 | 1.040 (1.015, 1.066) | 0.0002 |
| Q4 | 13,225 | 283,759.8 | 46.61 | 1.090 (1.064, 1.117) | < 0.0001 | 1.101 (1.071, 1.129) | < 0.0001 |
| p for trend |  |  |  | < 0.0001 |  | < 0.0001 |  |
| **Male subjects**  **(n = 95,044)** |  |  |  |  |  |  |  |
| Q1 | 7,406 | 169,708.2 | 43.64 | 1 |  | 1 |  |
| Q2 | 7,268 | 168,768.5 | 43.06 | 0.986 (0.946, 1.018) | 0.3790 | 0.981 (0.953, 1.010) | 0.2044 |
| Q3 | 7,603 | 166,433.7 | 45.68 | 1.044 (1.012, 1.078) | 0.0078 | 1.046 (1.013, 1.080) | 0.0104 |
| Q4 | 8,204 | 162,238.7 | 50.57 | 1.155 (1.119, 1.192) | < 0.0001 | 1.095 (1.059, 1.129) | < 0.0001 |
| p for trend |  |  |  | < 0.0001 |  | < 0.0001 |  |
| **Female subjects**  **(n = 67,189)** |  |  |  |  |  |  |  |
| Q1 | 5,038 | 122,208.4 | 41.22 | 1 |  | 1 |  |
| Q2 | 4,992 | 121,721.3 | 41.01 | 0.999 (0.956, 1.033) | 0.7257 | 1.027 (0.991, 1.064) | 0.1466 |
| Q3 | 5,200 | 120,527.7 | 43.14 | 1.045 (1.005, 1.086) | 0.0262 | 1.067 (1.030, 1.106) | 0.0003 |
| Q4 | 5,006 | 121,258.1 | 41.28 | 1.000 (0.962, 1.040) | 0.9938 | 1.107 (1.068, 1.147) | < 0.0001 |
| p for trend |  |  |  | 0.4203 |  | < 0.0001 |  |

^‡^including ICD-10 codes for hypertension with admission ≥ 1 or outpatient department visit ≥ 2, and at least one prescription of anti-hypertensive drug per year

^*^adjusted for age, sex, BMI, urine protein, mean hemoglobin level, CCI category, diabetes, dyslipidemia, NSAID use, smoking, exercise, income and family history of hypertension.

CV, coefficient of variation; BMI, body mass index; CCI, Charlson comorbidity index; Q, quartile; HR, hazard ratio; CI, confidence interval.

**Supplementary Table S6. Hazard ratio and 95% confidence interval for incidence of hypertension according to the quartiles of hemoglobin variability (Study outcomes with SBP ≥ 130 mmHg or DBP ≥ 80 mmHg, ACC/AHA criteria for hypertension, n = 19,871)**

| **All subjects**  **(n = 19,871)** | Events (n) | Follow-up duration (person-years) | Incidence rate  (per 1,000  person-years) | **HR (95% CI)** | | | |
| --- | --- | --- | --- | --- | --- | --- | --- |
|  |  |  |  | **Unadjusted** | **p-value** | **Adjusted^*^** | **p-value** |
| **CV** |  |  |  |  |  |  |  |
| Q1 | 3,123 | 24,725.8 | 126.31 | 1 |  | 1 |  |
| Q2 | 3,271 | 23,577.3 | 138.74 | 1.088 (1.036, 1.143) | 0.0007 | 1.085 (1.035, 1.140) | 0.0019 |
| Q3 | 3,299 | 23,279.8 | 141.71 | 1.108 (1.055, 1.163) | < 0.0001 | 1.117 (1.064, 1.174) | < 0.0001 |
| Q4 | 3,319 | 22,915.7 | 144.84 | 1.127 (1.073, 1.183) | < 0.0001 | 1.201 (1.142, 1.262) | < 0.0001 |
| p for trend |  |  |  | < 0.0001 |  | < 0.0001 |  |
| **SD** |  |  |  |  |  |  |  |
| Q1 | 3,066 | 24,970.3 | 122.79 | 1 |  | 1 |  |
| Q2 | 3,254 | 23,834.4 | 136.53 | 1.098 (1.045, 1.153) | 0.0002 | 1.074 (1.025, 1.124) | 0.0026 |
| Q3 | 3,321 | 23,136.3 | 143.54 | 1.149 (1.094, 1.206) | < 0.0001 | 1.132 (1.077, 1.189) | < 0.0001 |
| Q4 | 3,371 | 22,537.6 | 149.57 | 1.187 (1.131, 1.247) | < 0.0001 | 1.119 (1.140, 1.258) | < 0.0001 |
| p for trend |  |  |  | < 0.0001 |  | < 0.0001 |  |
| **VIM** |  |  |  |  |  |  |  |
| Q1 | 3,063 | 25,369.3 | 120.74 | 1 |  | 1 |  |
| Q2 | 3,176 | 24,028.8 | 132.17 | 1.082 (1.030, 1.137) | 0.0019 | 1.055 (1.007, 1.105) | 0.0238 |
| Q3 | 3,339 | 23,157.5 | 144.19 | 1.171 (1.115, 1.230) | < 0.0001 | 1.092 (1.043, 1.144) | 0.0002 |
| Q4 | 3,434 | 21,923.0 | 156.64 | 1.254 (1.194, 1.317) | < 0.0001 | 1.118 (1.068, 1.171) | < 0.0001 |
| p for trend |  |  |  | < 0.0001 |  | < 0.0001 |  |

^*^adjusted for age, sex, BMI, urine protein, mean hemoglobin level, CCI category, diabetes, dyslipidemia, NSAID use, smoking, exercise, income and family history of hypertension.

CV, coefficient of variation; SD, standard deviation; VIM, variability independent of the mean; BMI, body mass index; CCI, Charlson comorbidity index; Q, quartile; HR, hazard ratio; CI, confidence interval.

**Supplementary Table S7. Hazard ratio and 95% confidence interval for incidence of hypertension according to the quartiles of hemoglobin variability (Subjects with 4 ≥ health screenings in 2008 & 2009 as index year, n = 70,389)**

| **All subjects**  **(n = 70,389)** | Events (n) | Follow-up duration (person-years) | Incidence rate  (per 1,000  person-years) | **HR (95% CI)** | | | |
| --- | --- | --- | --- | --- | --- | --- | --- |
|  |  |  |  | **Unadjusted** | **p-value** | **Adjusted^*^** | **p-value** |
| **CV** |  |  |  |  |  |  |  |
| Q1 | 3,890 | 106,903.5 | 36.39 | 1 |  | 1 |  |
| Q2 | 3,817 | 106,816.4 | 35.73 | 0.982 (0.939, 1.027) | 0.4256 | 0.980 (0.937, 1.025) | 0.3847 |
| Q3 | 4,047 | 106,121.1 | 38.14 | 1.049 (1.004, 1.096) | 0.0341 | 1.051 (1.005, 1.099) | 0.0292 |
| Q4 | 4,187 | 106,294.3 | 39.39 | 1.084 (1.037, 1.132) | 0.0003 | 1.117 (1.067, 1.168) | < 0.0001 |
| p for trend |  |  |  | < 0.0001 |  | < 0.0001 |  |
| **SD** |  |  |  |  |  |  |  |
| Q1 | 3,840 | 107,322.0 | 35.78 | 1 |  | 1 |  |
| Q2 | 3,738 | 107,041.7 | 34.92 | 0.976 (0.933, 1.021) | 0.2887 | 0.971 (0.928, 1.016) | 0.2047 |
| Q3 | 4,087 | 105,997.5 | 38.56 | 1.079 (1.032, 1.127) | 0.0007 | 1.068 (1.021, 1.116) | 0.0039 |
| Q4 | 4,276 | 105,774.1 | 40.43 | 1.132 (1.083, 1.182) | < 0.0001 | 1.121 (1.072, 1.171) | < 0.0001 |
| p for trend |  |  |  | < 0.0001 |  | < 0.0001 |  |
| **VIM** |  |  |  |  |  |  |  |
| Q1 | 3,749 | 107,793.4 | 34.78 | 1 |  | 1 |  |
| Q2 | 3,762 | 107,187.7 | 35.10 | 1.009 (0.965, 1.056) | 0.6883 | 1.003 (0.958, 1.050) | 0.9058 |
| Q3 | 4,086 | 105,776.1 | 38.63 | 1.112 (1.064, 1.163) | < 0.0001 | 1.089 (1.041, 1.139) | 0.0002 |
| Q4 | 4,344 | 105,378.1 | 41.22 | 1.188 (1.137, 1.241) | < 0.0001 | 1.132 (1.082, 1.184) | < 0.0001 |
| p for trend |  |  |  | < 0.0001 |  | < 0.0001 |  |

^*^adjusted for age, sex, BMI, urine protein, mean hemoglobin level, CCI category, diabetes, dyslipidemia, NSAID use, smoking, exercise, income and family history of hypertension.

CV, coefficient of variation; SD, standard deviation; VIM, variability independent of the mean; BMI, body mass index; CCI, Charlson comorbidity index; Q, quartile; HR, hazard ratio; CI, confidence interval.

**Supplementary Table S8. Hazard ratio and 95% confidence interval for incidence of hypertension according to the quartiles of hemoglobin variability (Subjects with 4 ≥ health screenings in 2009 as index year, n = 39,591)**

| **All subjects**  **(n = 39,591)** | Events (n) | Follow-up duration (person-years) | Incidence rate  (per 1,000  person-years) | **HR (95% CI)** | | | |
| --- | --- | --- | --- | --- | --- | --- | --- |
|  |  |  |  | **Unadjusted** | **p-value** | **Adjusted^*^** | **p-value** |
| **CV** |  |  |  |  |  |  |  |
| Q1 | 3,890 | 106,903.5 | 36.39 | 1 |  | 1 |  |
| Q2 | 3,817 | 106,816.4 | 35.73 | 0.993 (0.934, 1.056) | 0.8233 | 1.013 (0.952, 1.077) | 0.6867 |
| Q3 | 4,047 | 106,121.1 | 38.14 | 1.084 (1.021, 1.151) | 0.0086 | 1.102 (1.037, 1.172) | 0.0018 |
| Q4 | 4,187 | 106,294.3 | 39.39 | 1.134 (1.068, 1.203) | < 0.0001 | 1.176 (1.104, 1.253) | < 0.0001 |
| p for trend |  |  |  | < 0.0001 |  | < 0.0001 |  |
| **SD** |  |  |  |  |  |  |  |
| Q1 | 3,840 | 107,322.0 | 35.78 | 1 |  | 1 |  |
| Q2 | 3,738 | 107,041.7 | 34.92 | 0.953 (0.896, 1.013) | 0.1248 | 0.973 (0.915, 1.036) | 0.3923 |
| Q3 | 4,087 | 105,997.5 | 38.56 | 1.088 (1.025, 1.156) | 0.0055 | 1.100 (1.035, 1.169) | 0.0023 |
| Q4 | 4,276 | 105,774.1 | 40.43 | 1.157 (1.091, 1.228) | < 0.0001 | 1.164 (1.094, 1.238) | < 0.0001 |
| p for trend |  |  |  | < 0.0001 |  | < 0.0001 |  |
| **VIM** |  |  |  |  |  |  |  |
| Q1 | 3,749 | 107,793.4 | 34.78 | 1 |  | 1 |  |
| Q2 | 3,762 | 107,187.7 | 35.10 | 0.994 (0.934, 1.057) | 0.8369 | 1.004 (0.943, 1.069) | 0.9011 |
| Q3 | 4,086 | 105,776.1 | 38.63 | 1.101 (1.036, 1.170) | 0.0018 | 1.110 (1.043, 1.181) | 0.0010 |
| Q4 | 4,344 | 105,378.1 | 41.22 | 1.239 (1.167, 1.314 | < 0.0001 | 1.191 (1.119, 1.267) | < 0.0001 |
| p for trend |  |  |  | < 0.0001 |  | < 0.0001 |  |

^*^adjusted for age, sex, BMI, GFR, mean hemoglobin level, CCI category, diabetes, dyslipidemia, NSAID use, smoking, exercise, income and family history of hypertension.

CV, coefficient of variation; SD, standard deviation; VIM, variability independent of the mean; BMI, body mass index; GFR, Glomerular filtration rate; CCI, Charlson comorbidity index; Q, quartile; HR, hazard ratio; CI, confidence interval.

**Supplementary Table S9. Hazard ratio and 95% confidence interval for incidence of hypertension according to the quartiles of hemoglobin variability (Subjects without hematopoietic disorder, cancer, chronic kidney disease, n = 51,490)**

| **All subjects**  **(n = 51,490)** | Events (n) | Follow-up duration (person-years) | Incidence rate  (per 1,000  person-years) | **HR (95% CI)** | | | |
| --- | --- | --- | --- | --- | --- | --- | --- |
|  |  |  |  | **Unadjusted** | **p-value** | **Adjusted^*^** | **p-value** |
| **CV** |  |  |  |  |  |  |  |
| Q1 | 3,513 | 98,113.6 | 37.59 | 1 |  | 1 |  |
| Q2 | 3,400 | 97,684.0 | 36.81 | 0.973 (0.928, 1.020) | 0.2501 | 0.987 (0.942, 1.034) | 0.5843 |
| Q3 | 3,626 | 96,992.3 | 39.43 | 1.045 (0.998, 1.095) | 0.0604 | 1.061 (1.013, 1.111) | 0.0119 |
| Q4 | 3,675 | 96,701.2 | 38.74 | 1.063 (1.015, 1.113) | 0.0010 | 1.081 (1.032, 1.132) | 0.0011 |
| p for trend |  |  |  | 0.0007 |  | < 0.0001 |  |
| **SD** |  |  |  |  |  |  |  |
| Q1 | 3,485 | 98,397.5 | 37.06 | 1 |  | 1 |  |
| Q2 | 3,337 | 97,295.5 | 36.53 | 0.969 (0.924, 1.016) | 0.1932 | 0.982 (0.937, 1.029) | 0.4446 |
| Q3 | 3,623 | 97,484.6 | 39.03 | 1.051 (1.003, 1.101) | 0.0362 | 1.056 (1.008, 1.106) | 0.0212 |
| Q4 | 3,769 | 96,313.5 | 39.95 | 1.107 (1.057, 1.159) | < 0.0001 | 1.083 (1.034, 1.134) | 0.0007 |
| p for trend |  |  |  | < 0.0001 |  | < 0.0001 |  |
| **VIM** |  |  |  |  |  |  |  |
| Q1 | 3,465 | 98,352.8 | 36.26 | 1 |  | 1 |  |
| Q2 | 3,368 | 97,965.8 | 36.54 | 0.976 (0.931, 1.024) | 0.3241 | 0.987 (0.942, 1.035) | 0.6001 |
| Q3 | 3,600 | 97,012.1 | 38.61 | 1.055 (1.007, 1.105) | 0.0248 | 1.054 (1.006, 1.104) | 0.0262 |
| Q4 | 3,781 | 96,160.4 | 41.21 | 1.118 (1.068, 1.171) | < 0.0001 | 1.078 (1.029, 1.128) | 0.0015 |
| p for trend |  |  |  | < 0.0001 |  | < 0.0001 |  |

^*^adjusted for age, sex, BMI, urine protein, mean hemoglobin level, CCI category, diabetes, dyslipidemia, NSAID use, smoking, exercise, income and family history of hypertension.

CV, coefficient of variation; SD, standard deviation; VIM, variability independent of the mean; BMI, body mass index; CCI, Charlson comorbidity index; Q, quartile; HR, hazard ratio; CI, confidence interval.

**Supplementary Table S10. Hazard ratio and 95% confidence interval for incidence of hypertension according to the quartiles of hemoglobin variability (Subjects with CCI = 0, n = 60,718)**

| **All subjects**  **(n = 60,718)** | Events (n) | Follow-up duration (person-years) | Incidence rate  (per 1,000  person-years) | **HR (95% CI)** | | | |
| --- | --- | --- | --- | --- | --- | --- | --- |
|  |  |  |  | **Unadjusted** | **p-value** | **Adjusted^*^** | **p-value** |
| **CV** |  |  |  |  |  |  |  |
| Q1 | 4,315 | 114,798.8 | 37.59 | 1 |  | 1 |  |
| Q2 | 4,200 | 114,103.9 | 36.81 | 0.980 (0.940, 1.023) | 0.3594 | 0.990 (0.949, 1.032) | 0.6237 |
| Q3 | 4,454 | 112,971.8 | 39.43 | 1.051 (1.008, 1.096) | 0.0202 | 1.060 (1.017, 1.105) | 0.0061 |
| Q4 | 4,399 | 113,558.0 | 38.74 | 1.032 (0.989, 1.076) | < 0.0001 | 1.084 (1.038, 1.131) | 0.0002 |
| p for trend |  |  |  | 0.0167 |  | < 0.0001 |  |
| **SD** |  |  |  |  |  |  |  |
| Q1 | 4,264 | 115,066.8 | 37.06 | 1 |  | 1 |  |
| Q2 | 4,156 | 113,774.1 | 36.53 | 0.986 (0.945, 1.029) | 0.5299 | 0.990 (0.950, 1.033) | 0.6454 |
| Q3 | 4,433 | 113,566.2 | 39.03 | 1.055 (1.012, 1.100) | 0.0124 | 1.054 (1.011, 1.099) | 0.0137 |
| Q4 | 4,515 | 113,025.4 | 39.95 | 1.080 (1.035, 1.126) | 0.0003 | 1.091 (1.046, 1.138) | < 0.0001 |
| p for trend |  |  |  | < 0.0001 |  | < 0.0001 |  |
| **VIM** |  |  |  |  |  |  |  |
| Q1 | 4,184 | 115,389.7 | 36.26 | 1 |  | 1 |  |
| Q2 | 4,180 | 114,395.1 | 36.54 | 1.003 (0.941, 1.069) | 0.6967 | 1.013 (0.971, 1.057) | 0.5509 |
| Q3 | 4,376 | 113,350.6 | 38.61 | 1.066 (1.022, 1.113) | 0.0029 | 1.055 (1.012, 1.101) | 0.0126 |
| Q4 | 4,628 | 112,297.1 | 41.21 | 1.139 (1.093, 1.188) | < 0.0001 | 1.099 (1.053, 1.146) | < 0.0001 |
| p for trend |  |  |  | < 0.0001 |  | < 0.0001 |  |

^*^adjusted for age, sex, BMI, urine protein, mean hemoglobin level, CCI category, diabetes, dyslipidemia, NSAID use, smoking, exercise, income and family history of hypertension.

CV, coefficient of variation; SD, standard deviation; VIM, variability independent of the mean; BMI, body mass index; CCI, Charlson comorbidity index; Q, quartile; HR, hazard ratio; CI, confidence interval.

**Supplementary Table S11. Hazard ratio and 95% confidence interval for incidence of hypertension according to the quartiles of hemoglobin variability (Subjects with absent of urine protein, n = 93,579)**

| **All subjects**  **(n = 93,579)** | Events (n) | Follow-up duration (person-years) | Incidence rate  (per 1,000  person-years) | **HR (95% CI)** | | | |
| --- | --- | --- | --- | --- | --- | --- | --- |
|  |  |  |  | **Unadjusted** | **p-value** | **Adjusted^*^** | **p-value** |
| **CV** |  |  |  |  |  |  |  |
| Q1 | 7,103 | 174,211.0 | 40.77 | 1 |  | 1 |  |
| Q2 | 7,011 | 173,310.7 | 40.45 | 0.993 (0.961, 1.026) | 0.6663 | 1.007 (0.974, 1.040) | 0.6954 |
| Q3 | 7,377 | 171,260.7 | 43.07 | 1.058 (1.024, 1.093) | 0.0007 | 1.063 (1.029, 1.098) | 0.0003 |
| Q4 | 7,346 | 170,745.2 | 43.02 | 1.056 (1.023, 1.091) | 0.0010 | 1.087 (1.051, 1.123) | < 0.0001 |
| p for trend |  |  |  | < 0.0001 |  | < 0.0001 |  |
| **SD** |  |  |  |  |  |  |  |
| Q1 | 7,048 | 174,590.8 | 40.37 | 1 |  | 1 |  |
| Q2 | 6,952 | 173,547.7 | 40.06 | 0.993 (0.960, 1.026) | 0.6634 | 1.003 (0.971, 1.037) | 0.8487 |
| Q3 | 7,322 | 171,490.9 | 42.70 | 1.059 (1.025, 1.094) | 0.0006 | 1.053 (1.019, 1.088) | 0.0020 |
| Q4 | 7,515 | 169,898.2 | 44.23 | 1.098 (1.062, 1.134) | < 0.0001 | 1.093 (1.057, 1.129) | < 0.0001 |
| p for trend |  |  |  | < 0.0001 |  | < 0.0001 |  |
| **VIM** |  |  |  |  |  |  |  |
| Q1 | 6,974 | 175,184.6 | 39.81 | 1 |  | 1 |  |
| Q2 | 6,959 | 173,303.5 | 40.15 | 1.009 (0.976, 1.043) | 0.5848 | 1.020 (0.986, 1.054) | 0.2462 |
| Q3 | 7,280 | 171,899.3 | 42.35 | 1.065 (1.031, 1.101) | 0.0002 | 1.052 (1.018, 1.087) | 0.0027 |
| Q4 | 7,624 | 169,140.2 | 45.07 | 1.135 (1.099, 1.172) | < 0.0001 | 1.094 (1.059, 1.131) | < 0.0001 |
| p for trend |  |  |  | < 0.0001 |  | < 0.0001 |  |

^*^adjusted for age, sex, BMI, mean hemoglobin level, CCI category, diabetes, dyslipidemia, NSAID use, smoking, exercise, income and family history of hypertension.

CV, coefficient of variation; SD, standard deviation; VIM, variability independent of the mean; BMI, body mass index; CCI, Charlson comorbidity index; Q, quartile; HR, hazard ratio; CI, confidence interval.

**Supplementary Table S12. Hazard ratio and 95% confidence interval for incidence of hypertension according to the quartiles of hemoglobin variability (Subjects with GFR** ≥ **90 at the final health screening, n = 43,942)**

| **All subjects**  **(n = 43,942)** | Events (n) | Follow-up duration (person-years) | Incidence rate  (per 1,000  person-years) | **HR (95% CI)** | | | |
| --- | --- | --- | --- | --- | --- | --- | --- |
|  |  |  |  | **Unadjusted** | **p-value** | **Adjusted^*^** | **p-value** |
| **CV** |  |  |  |  |  |  |  |
| Q1 | 3,178 | 82,948.3 | 38.31 | 1 |  | 1 |  |
| Q2 | 3,145 | 82,616.7 | 38.07 | 0.995 (0.947, 1.045) | 0.8267 | 1.000 (0.952, 1.051) | 0.9915 |
| Q3 | 3,299 | 81,935.6 | 40.26 | 1.053 (1.003, 1.105) | 0.0383 | 1.059 (1.009, 1.112) | 0.0210 |
| Q4 | 3,310 | 82,214.4 | 40.26 | 1.052 (1.002, 1.105) | 0.0404 | 1.089 (1.036, 1.145) | 0.0008 |
| p for trend |  |  |  | 0.0077 |  | 0.0001 |  |
| **SD** |  |  |  |  |  |  |  |
| Q1 | 3,126 | 82,978.9 | 37.67 | 1 |  | 1 |  |
| Q2 | 3,136 | 82,960.4 | 37.80 | 1.004 (0.956, 1.055) | 0.8677 | 0.998 (0.950, 1.049) | 0.9405 |
| Q3 | 3,265 | 81,938.9 | 39.85 | 1.060 (1.009, 1.113) | 0.0206 | 1.047 (0.997, 1.100) | 0.0679 |
| Q4 | 3,405 | 81,836.8 | 41.61 | 1.107 (1.055, 1.162) | < 0.0001 | 1.097 (1.044, 1.152) | 0.0002 |
| p for trend |  |  |  | < 0.0001 |  | < 0.0001 |  |
| **VIM** |  |  |  |  |  |  |  |
| Q1 | 3,088 | 83,509.3 | 36.98 | 1 |  | 1 |  |
| Q2 | 3,105 | 82,625.2 | 37.58 | 1.017 (0.968, 1.069) | 0.4952 | 1.009 (0.960, 1.060) | 0.7319 |
| Q3 | 3,223 | 82,253.5 | 39.18 | 1.062 (1.011, 1.116) | 0.0173 | 1.027 (0.978, 1.080) | 0.2852 |
| Q4 | 3,516 | 81,327.0 | 43.23 | 1.173 (1.118, 1.231) | < 0.0001 | 1.106 (1.053, 1.161) | < 0.0001 |
| p for trend |  |  |  | < 0.0001 |  | < 0.0001 |  |

^*^adjusted for age, sex, BMI, GFR, mean hemoglobin level, CCI category, diabetes, dyslipidemia, NSAID use, smoking, exercise, income and family history of hypertension.

CV, coefficient of variation; SD, standard deviation; VIM, variability independent of the mean; BMI, body mass index; GFR, Glomerular filtration rate; CCI, Charlson comorbidity index; Q, quartile; HR, hazard ratio; CI, confidence interval.

**Supplementary Figure S1. Hazard ratio and 95% confidence interval for incidence of hypertension of hemoglobin variability (CV) in female subjects dividing by 50 years**

CV, coefficient of variation; HR, hazard ratio; CI, confidence interval; Q, quartile.


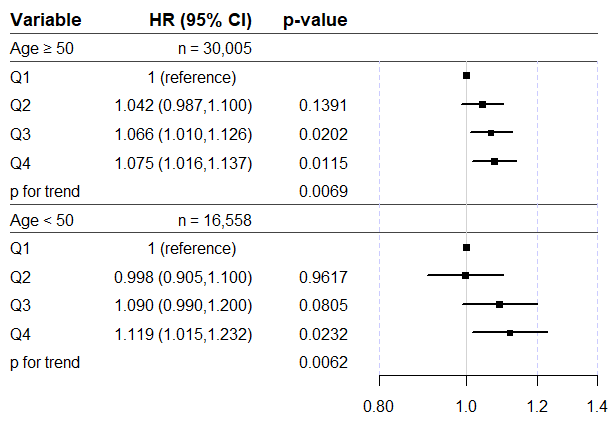

Supplement: Supplementary file 1 — Supplementary Information. [file 41598_2020_69022_MOESM1_ESM.docx]
